# Estimate cost of providing methadone maintenance treatment at a methadone clinic in Nairobi Kenya: direct costs

**DOI:** 10.11604/pamj.2021.38.84.21991

**Published:** 2021-01-26

**Authors:** Brenda Mogaka, Sarah Kanana Kiburi, Mirriam Mutinda, Maxwel Kendagor

**Affiliations:** 1 Department of Pharmacy, Ngara Medically-Assisted Therapy (MAT) Clinic, Nairobi, Kenya,; 2 Department of Psychiatry, Ngara Medically-Assisted Therapy (MAT) Clinic, Nairobi, Kenya,; 3 Kenya Programs, University of Maryland, Maryland, USA

**Keywords:** Cost, methadone maintenance treatment, Nairobi

## Abstract

Methadone maintenance treatment is reported as cost-effective in treatment of opioid use disorder. Estimated cost of providing methadone varies widely in different regions but there is no data regarding cost of methadone treatment in Kenya. The aim of this study was to estimate the cost of methadone maintenance treatment at a methadone maintenance treatment clinic in Nairobi, Kenya from the perspective of the government, implementing partner and the clients. Data was collected for the period of February 2017 to September 2018 for 700 enrolled clients. The cost of providing methadone treatment was estimated as the sum of salaries, laboratory test, methadone and other commodities costs. The outcome was daily cost of methadone per client. The costs are given in Kenya Shillings (Ksh). The cost of treating one client is approximately Ksh. 149 (US$1.49) per day which amounts to Ksh 4500 (US$ 45) per month. This is from the estimated direct costs such as salaries which accounted for 86.4%, methadone 9.6%, tests and other consumables at 4%. The estimated average dose per patient per day is 60mg.This excludes indirect costs such as capital and set up cost, maintenance cost, training, drug import and distribution and other bills. The findings of this study show that the estimate cost of providing methadone at Nairobi, Kenya is comparable to that in other centers. This can help to inform policy makers on continued provision of methadone treatment in the country.

## Introduction

Opioid use is prevalent worldwide with 1.2% of people aged 15-64 years reporting to have used opioids in 2018 [1]. There is limited research on opioid use in Africa but it has been shown to be an emerging problem [1]. In Kenya, the prevalence in the general population aged 15-65 years is less than 0.1% in 2017 [2]. Opioid use accounts for most of the negative effects of substance use [1]. These include; increased risk for infections such as HIV and hepatitis, increased involvement in crime, unemployment and increased mortality [3]. In addition there is significant economic burden associated with opioid use due to cost of hospital visits, loss of productive work [4]. In Kenya, HIV prevalence rate among people with injection drug use is three times that in the general population [5]. Opioid substitution therapy (OST) is treatment used for treatment of opioid use disorder. Methadone maintenance treatment (MMT) refers to use of methadone for OST. Methadone acts on µ opioid receptors resulting in alleviation of withdrawal symptoms with minimal risk of tolerance and reduction in craving and compulsive opioid use. In addition it is the OST that has wider evidence base and more availability [3,4]. Opioid substitution therapy has several benefits which reduce burden of opioid use disorder [3], and is recommended as part of pharmacotherapy for opioid use disorder treatment [6]. However despite the reported benefits, OST use globally is suboptimal. One study reported that in 2014, 85% of United Nations members reported low or medium OST coverage while 5% reported no coverage of services. Additionally this availability is less in LMICs [7].

The government through support of partners has set up public MMT clinics as part of the national harm reduction strategy for HIV prevention since December 2014 [3]. In Kenya MMT is part of the recommended pharmacological treatment for opioid use disorders in the national treatment guidelines provided by the Ministry of health [5]. Currently there are eight public MMT clinics in Kenya. Prior to this, treatment for opioid use disorder was usually short-lived detoxification based in private facilities which had limited availability and was associated with high relapse rates [3]. A systematic review showed that MMT is economically advantageous for treatment of opioid use disorder [4]. A qualitative study among people on MMT in Kenya reported several benefits such as more engagement in treatment for HIV care, MMT was reported as more available and effective treatment compared to rehabilitation centres and the MMT program was perceived as a source of aspiration and hope for the community that supported recovery [3]. Estimated cost of providing methadone varies widely in different regions [7], but there is no supporting data to assess the cost of methadone treatment in Kenya. As at the time of this publication methadone is the only OST available in public funded clinics in Kenya. The World Health Organisation (WHO) recommends assessment of local needs including treatment and care delivery programs to help inform allocation of resources and designing of local treatment system for substance use disorders [6]. The aim of this study was therefore to estimate the cost of methadone treatment at a MMT clinic in Nairobi, Kenya. This will inform policy regarding the implementation of MMT in Kenya.

## Methods

**Study location and site description:** this study was based at Ngara MMT clinic which is based in Nairobi, Kenya and has been in operation since February 2017. In addition to methadone, other services offered at the clinic include psychosocial treatment, management of other substance use disorders, testing and treatment for other co-occurring medical illnesses such as HIV, hepatitis and tuberculosis and psychiatric disorders.

**Data collection and analysis:** data was collected for the period of February 2017 to September 2018 for 700 clients who had been enrolled in the MMT program during that period. The costing was done from provider perspective whereby only direct costs was estimated based on the sum of salaries for the personnel, laboratory tests (including accessories such as testing kits), methadone and other commodities costs. Data was based on document analysis for operational costs at the clinic. No individual interviews were conducted. Methadone cost was estimated using an average of 60mg per client per day. Indirect costs were excluded due to limitations in getting the data by the authors. Data on daily cost to client was obtained by assessing records on the reported amount of money used to purchase heroin prior to enrolment in the program. The costs are given in Kenya Shillings (Ksh) and the amount in US Dollars indicated in brackets for comparison with other countries at the discussion.

## Results

**Figure 1:**
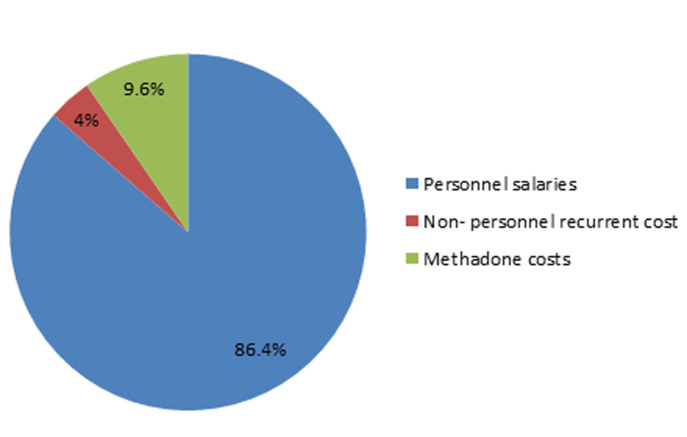
percentage of direct cost items to total cost of methadone maintenance treatment provision

The sum of the total cost of methadone treatment per year was Ksh. 37,619,300 (US$ 376193). The average daily cost of methadone treatment was US$ 1.49 per individual with average personnel cost constituting most of the cost (86.4%) while methadone cost comprised a tenth of the total. The other non-personnel recurrent costs comprised 4% of the direct costs with majority of this cost being from cost of dispensing cups. This is shown in more details in Table 1 and Figure 1.

**Table 1:** summary of daily cost of methadone treatment based on direct costs

Item	Cost per month in Ksh (US$)	Cost per month in Ksh (US$)
**Personnel (salaries)**	2,710,000	32,520,000
**Non-personnel recurrent**	124,940	1,499,300
Dispensing cups	105,000	1,260,000
Urine cups	5000	60,000
Urine sticks	5000	60,000
Hepatitis B and C testing Kits	4200	50,400
Needles and syringes	4700	56,400
Cotton wool	1040	12,500
Methadone	300,000	3600000
**Total**	3,130340 (31,303.40)	37619300 (376,193)
Cost per client	4471 (44.71)	53,742 (537.42)
Average cost per client per day	149 (1.49)	149 (1.49)

**Estimation of costs to client:** the transport cost spent by the client to come daily is about Ksh. 200 (US$ 2) per day; this is compared to Ksh 1000 (US$ 10) they used to spend daily on buying heroin saving 80% of their income.

## Discussion

The average cost of proving methadone per client per day was found to be Ksh 149 (US$1.49). This is almost similar to the estimate cost in lower and middle income countries such as Vietnam and Indonesia which was $1.01 and US$1.11 respectively [7]. However, studies done in high-income countries have reported higher costs [5,8] and in China the cost ranged between US$ 0.33-0.36 [7,8]. Reasons for the varying cost could be due to differences in the costs included and how the costs are reported well as the different years of study. This shows role of having local studies to assess cost in each country [6]. In this study, personnel cost accounted for 86% of the total cost of treatment. This is higher compared to other studies. For example, in Vietnam, labor was 66% of direct cost of methadone treatment [9] while in Canada urine sample costs were highest at 46.7% followed by pharmacy costs at 39.8% [10]. This may be influenced by different remuneration and labor wages in different countries, other services provided at the clinic hence more staff as well difference in calculation of the costs. Methadone cost was approximately a tenth (9.6%) of the cost. Findings from other studies vary for example in Canada, methadone cost was 3.8% of average daily cost of MMT while in Vietnam it comprised 13% [9,10]. This difference in cost may be influenced by difference in average dose of methadone used for estimation of cost, and difference in type of methadone used since different types have varying costs [7]. In addition the studies have been done in different years.

**Study limitations:** first, this study did not include indirect cost such as capital which may have affected the average cost as they have been shown to account for significant percentage of total costs [9]. Secondly, this study did not include cost incurred by clients enrolled in the program which may significantly influence the cost of MMT. Additionally, the findings are derived from one facility hence may not be generalizable since other MMT clinics may have a different set up and policies.

## Conclusion

The findings of this study show that the estimate cost of providing methadone at a MMT clinic in Nairobi Kenya is comparable to that in other centers globally. This information is important in that it can help to inform policy makers on the average cost given the plan of expansion of methadone treatment in the country. This will facilitate management of patients with opioid use disorders. Based on the findings of this study, the following recommendations are suggested. There is need to conduct studies the other MMT facilities in the country to assess the cost of methadone provision and have comparison of costs. In addition, future studies need to have a societal perspective of costing and include other costs such as client costs (travel cost to clinic, cost of employment time lost) or societal benefits (e.g. reduction in crime, reduction in infections, employment benefits) this will give a comprehensive cost-effectiveness analysis and provide the economic benefit of MMT as an intervention. The findings in this study suggest that the majority of cost was from salaries of personnel. This shows that if MMT is integrated in the general hospitals set up and use the existing staff instead of employing more staff in separate MMT clinic, this can reduce the total cost. In addition, MMT integrated within the general hospital set up will ensure that treatment for opioid use disorder is initiated early with referrals where necessary and treatment for any comorbid medical or psychiatric conditions. This will help alleviate the stigma associated with MMT clinics situated separately [6].

### What is known about this topic

- Opioid use disorder is associated with several negative effects;
- Provision of opioid substitution therapy with methadone reduces the risks associated with opioid use and opioid use disorder;
- Methadone treatment is cost-effective for treatment of opioid use disorder.

### What this study adds

- The estimate cost of offering methadone treatment at a methadone maintenance treatment clinic in Nairobi, Kenya based on direct costs. The cost is found to be comparable to cost in other developing countries;
- This study found that majority of the cost was from personnel remuneration with methadone cost comprising a tenth of the cost. This is important as it gives information on allocation of resources;
- This information is important to inform policy on methadone treatment in Kenya to improve treatment of opioid use disorders. Additionally it forms a basis on which further research can be done to assess cost-effectiveness of methadone maintenance treatment in Kenya.
